# Electrides: Emerging electronic materials for catalysis

**DOI:** 10.1016/j.fmre.2024.11.026

**Published:** 2024-12-09

**Authors:** Fangkun Sun, Zhilin Guo, Yangfan Lu, Jiang Li, Tian-Nan Ye, Hideo Hosono, Jiazhen Wu

**Affiliations:** a Department of Materials Science and Engineering, Southern University of Science and Technology, Shenzhen 518055, China; b Guangdong Provincial Key Laboratory of Functional Oxide Materials and Devices, Southern University of Science and Technology, Shenzhen 518055, China; c Institute of Innovative Materials, Southern University of Science and Technology, Shenzhen 518055, China; d College of Materials Science and Engineering, National Engineering Research Center for Magnesium Alloys, Chongqing University, Chongqing 400044, China; e MDX Research Center for Element Strategy, International Research Frontiers Initiative, Tokyo Institute of Technology, Yokohama 226-8503, Japan; f Frontiers Science Center for Transformative Molecules, School of Chemistry and Chemical Engineering, Shanghai Jiao Tong University, Shanghai 200240, China

**Keywords:** Electrides, Low work function, Electron-donor, Heterogeneous catalysis, Chemical reactions

## Abstract

Electrides are emerging materials that exhibit a unique electronic structure, where electrons, unbound to specific atomic nuclei, act as anions within periodic lattice vacancies. The most characteristic feature of an electride is its low work function (Φ_WF_ < ∼3.5 eV), comparable to alkali metals, making it a highly promising electron donor in chemical reactions. In this paper, we summarized recent applications of variable electrides in various reactions, especially as catalysts. We thoroughly explored their unique behaviors and demonstrated their broad applicability in multiple reactions, such as selective hydrogenation, carbon-carbon coupling reactions, and electrocatalysis. In addition, we discussed the current challenges of electrides with active electron anions and highlighted their substantial potential for application in future advancements. This review provides fundamental guidance for utilizing high-performance electride-based materials in various chemical reactions, mainly focusing on heterogeneous catalysis.

## 1 Introduction

The design and application of catalysts have always been a cornerstone of chemical reactions and industrial manufacturing, playing a critical role in synthesizing natural products, medicine, polymers and essential accessories. Their importance lies in accelerating reactions without consumption, enabling more efficient and cost-effective processes. Traditional catalysts based on metal complexes are of continuous interest due to their high catalytic efficiency and selectivity under mild conditions and their versatility in tunable ligands and metal centers. However, many of them undergo a homogeneous catalytic mechanism, posing challenges in recovery and recycling. The complex synthetic procedures for metal complexes also limit their practicality for large-scale industrial applications.

In recent years, with the discovery of new materials, the focus on sustainability and green chemistry has propelled the importance of inorganic catalysts, also known as heterogeneous catalysts. This catalytic system, including metal oxides, carbides, nitrides, sulfides and metal nanoparticles, has garnered significant attention. As a catalytic active center, metal catalysts can be deposited onto a range of supports, for instance, refractory oxides like SiO_2_, Al_2_O_3_, or TiO_2_ [1], carbon and zeolite [2,3], metal-organic frameworks (MOFs) [4], covalent organic frameworks (COFs) [5], and polyoxometalates (POMs) [6], to realize the dispersion of the metal active sites and improve the stability, necessitating the prevention of irreversible aggregation [7]. In this case, the interaction between the active metal substrate and support, which may lead to the rearrangement of surface charges, is associated with the catalytic performance. The direction and extent of charge transfer are determined by the difference in Fermi levels between the active metal and the support, ultimately striving to achieve an equilibrium in electronic chemical potential, thereby affecting the absorption and activation of the reactant molecules [1,8]. Therefore, the rational design of heterogeneous catalysts generally focuses on developing stable intrinsic materials bearing active catalytic metal centers with high surface area, as well as constructing supports with strong electronic modification.

A particularly intriguing development in this field is the emergence of electrides, especially inorganic electrides [[9], [10], [11], [12]]. These are novel materials where electrons are periodically localized in interstitial spaces and serve as anions. The loosely bound electrons in the crystal endow electrides with a wide range of distinctive properties, including magnetism, abundant topological states [13], superconductivity [14], high electron mobility and polarizability, which have open avenues for applications in heterogeneous catalysis, batteries, non-linear optics, energy conversion and storage [15,16]. Electride materials have gained significant attention as novel inorganic catalysts due to their high carrier density and low work function, both of which are highly advantageous for catalytic processes. These unique characteristics enable efficient electron transfer, making electrides highly effective in various catalytic reactions. Transition metals such as Co, Fe, Cu, Ru, Pd, and Pt can be easily integrated into electrides either by incorporation within the lattice or by being dispersed on the surface.

**Fig. 1:**
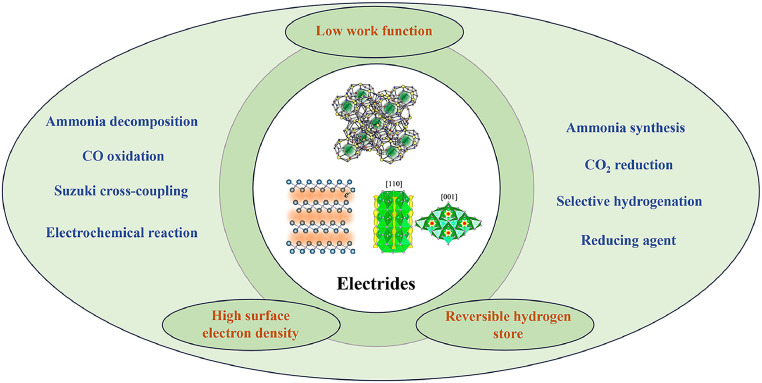
**Overview of various chemical reactions using electride-based catalysts**.

In the early stages of research, electrides were primarily explored as single-electron donor reagents in specific chemical reactions, such as the pinacol coupling reaction applied in aqueous media [17]. Their catalytic potential remained largely underexplored until a breakthrough in 2012 with the discovery of Ru-loaded electrides for ammonia synthesis [18]. Since then, electrides have attracted growing interest, with research efforts focusing on the development of novel, stable electride materials and their application in a broad range of catalytic processes (Fig. 1), such as hydrogenation reactions [19], CO*_x_* activation [20], Suzuki coupling [21], and electrocatalytic hydrogen evolution reactions [22]. A comprehensive review by Hosono et al. has provided a foundational understanding of the chemical properties and applications of electride materials [15]. In this paper, we further explore recent advancements in the catalytic applications of electrides, focusing on their role in small gas molecule activation, organic substrate transformations, and electrochemical and photochemical processes. We aim to provide a thorough exploration of the underlying mechanisms driving electride catalysis and offer perspectives on future research directions.

## 2 Electride materials

### 2.1 Organic electrides

Organic electrides are the earliest prototypes of electrides. The concept of an electride may originate from solvated electrons. It is observed that there is a dramatic color change when alkali metals dissolve in liquid ammonia, where electrons from alkali metals become solvated. This phenomenon has inspired and led to a significant breakthrough in chemistry: the first discovery of an electride compound by Dye et al. in the 1980s, featuring a Cs center with an 18-crown-6 ligand [9]. Since then, a variety of electrides based on alkali metal (K, Rb, Li, etc.) and crown ether or cryptand ligands have been synthesized and characterized as organic electrides [[23], [24], [25]]. Recent studies have demonstrated that employing strong reducing agents is an effective method for obtaining organic electrides. For example, a room-temperature-stable Mg electride with bipyridine ligand has been successfully isolated through Ni(II) reduction [26]. A *K*^+^[LiN(SiMe_3_)_2_]*e*^−^ electride was prepared by ball-milling of K metal and a Li complex. Additionally, another Ca electride has been synthesized using a similar ligand system, indicating that strong Lewis base donor ligands are effective stabilizers for organic electrides [27]. Initially, the presence of anionic electrons was identified by noise-level electron density at the typical anionic site from single crystal X-ray diffraction [28]. Subsequently, electron paramagnetic resonance (EPR) and magnetic susceptibility measurements confirmed the electride nature, revealing strong single-electron spin or non-diamagnetic behaviors, respectively. Alongside experimental syntheses, theoretical investigations have predicted other organic electrides, such as M_2_^•+^TCNQ^•−^ (*M* = Li, Na, K) [29] and Li@Calix[3]Pyrrole [30]. However, the practical use of organic electrides is still limited due to their extreme sensitivity to air and moisture, necessitating synthesis and handling under strictly controlled inert atmospheres, often at cryogenic temperatures. These stability challenges hinder their broader application beyond specialized laboratory environments.

### 2.2 Inorganic ionic electrides

**Fig. 2:**
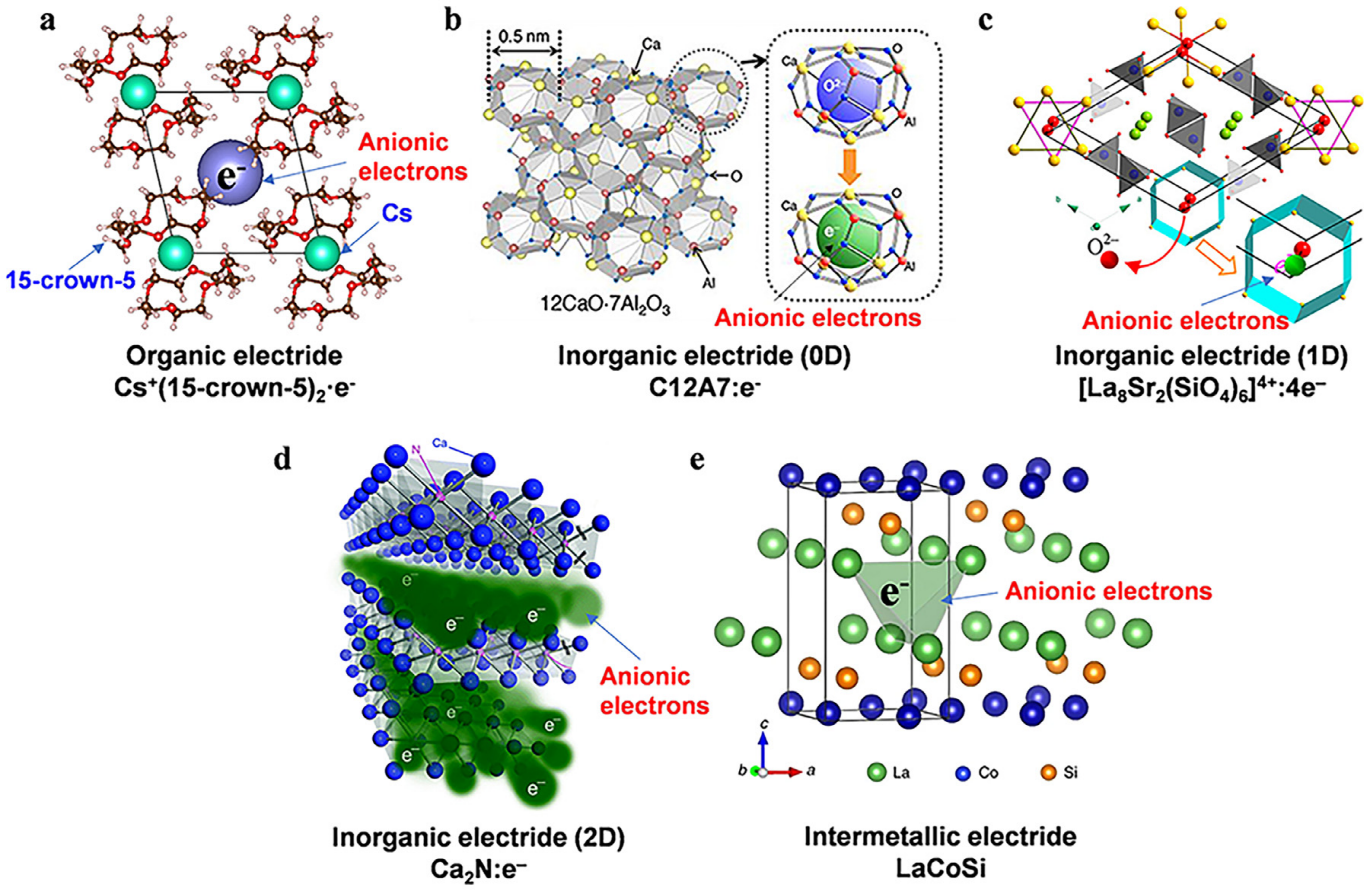
**Crystal structures of various types of electride materials**.

The first inorganic electride, C12A7:*e*^−^, was discovered by Hosono's group in 2003 [10]. The mother material 12CaO·7Al_2_O_3_ (C12A7) was known as a major component of alumina cement. Its structure contains twelve positively charged cages ([Ca_24_Al_28_O_64_]^4+^) per unit cell, with oxygen ion (O^2−^) accommodated in 1/6 of the cages. The oxygen ion is weakly bound by the cage framework due to the oversized cages. The O^2−^ within the cages can be replaced by various anions and, in an extreme case, anionic electrons, forming the first chemically and thermally stable electride C12A7:*e*^−^. These in-cage anionic electrons within the crystal lattice are a characteristic feature of zero-dimensional (0D) electrides (Fig. 2). C12A7:*e*^−^ exhibits a low work function (Φ_WF_) of 2.4 eV, high conductivity at room temperature of 1380 S·cm^−1^, and high electron concentration of 2.3×10^21^ cm^−3^, making it a promising material in electron emitter, cathode, optical device, catalyst support and other fields. Generally, C12A7:*e*^−^ is prepared under a reductive atmosphere using Ca/Ti or CaH_2_ to remove the in-cage oxygen ions from C12A7. The synthetic strategy involving metal hydrides can also be applied to other oxides with potentially reducible oxygen sites within the crystal lattice, enabling the preparation of oxide electrides such as La_8_Sr_2_(SiO_4_)_6_:4e^–^, La_1.5_Sr_0.5_Ga_3_O_7.25_, [Y_2_Ti_2_O_6_]^2+^:2e^−^ and BaAl_2_O_4–_*_x_*H*_y_* [[31], [32], [33]]. Interestingly, the anionic electrons in La_8_Sr_2_(SiO_4_)_6_:4e^–^ are arranged in a one-dimensional (1D) channel, making it a 1D electride (Fig. 2). Similarly, Wang et al. predicted and experimentally validated new 1D phosphide electrides such as Sr_5_P_3_ [34] and Ba_5_P_3_ [35]. Although density functional calculations using generalized gradient approximation predict Sr_5_P_3_ as a metal, electrical conductivity measurements reveal semiconductor properties characterized by a distinct band gap, indicating that the newly synthesized Sr_5_P_3_ is an ideal 1D electride with unpaired electrons in the half-filled band. For Ba–P or Sr–P systems, it was shown that the ionic bond (Coulomb attraction) between the anionic electrons and neighboring cations enhanced the stability of the crystal structure, while the Coulomb repulsion between the anionic electrons decreased the possibility of the appearance of high-dimensional electrides.

In addition to the reduction method, electrides were discovered in many intrinsic non-stoichiometric compounds (in the sense of charge neutrality between cations and anions), such as Ca_2_N, which can be formally written as [Ca^2+^]_2_N^3–^:e^–^. The interstitial anionic electrons in the Ca_2_N electride are fully delocalized within the [Ca_2_N]^+^ layer, resulting in a two-dimensional (2D) electride structure (Fig. 2). More 2D electrides have been discovered, typically consisting of alkaline-earth or rare-earth metals combined with p-block elements (C, N, Cl, P, As, Sb) ([[36], [37], [38]]) and even beyond the 2:1 ratio (e.g., YCl, ScCl) [39]. Hydride electrides, such as LaH_2_ and CeH_2_ [40], have also been reported. Most recently, a 2D electride, BaCu, has been synthesized, where Cu acts as a metal anion and the anionic electrons are delocalized within the interlayer space of the BaCu framework [41].

As mentioned earlier, electrides can be categorized based on the structural characteristics of their anionic electrons. In addition to 0D, 1D, and 2D electrides, three-dimensional (3D) electrides, where anionic electrons are dispersed throughout the entire crystal structure, have also been theoretically predicted. A typical example of this is Rb_3_O [42]. Fig. 2 illustrates some representative crystal structures of electrides to demonstrate the dimensionalities, and Table 1 summarizes selected electrides and their work functions.

**Table 1:** **Selected inorganic electrides and their properties**.

Examples	Dimensions of electrides	Work function (eV)	Ref
[Ca_24_Al_28_O_64_]^4+^:4e^‒^	0D	2.4	[43]
Ba_4_Al_5_:e^‒^	0D	2.09	[44]
LaCu_0.67_Si_1.33_	0D	3.5	[19]
[Y_5_Si_3_]^0.79+^:0.79e^–^	1D	3.5	[11]
[La_8_Sr_2_(SiO_4_)_6_]^4+^:4e^‒^	1D	–	[31]
Sr_5_P_3_	1D	3.5	[34]
Sr_3_CrN_3_	1D	2.14 a	[36]
Sr_2_N:*e*^−^	2D	3.2 a	[37]
Ca_2_N:e^−^	2D	2.6	[45]
[Y_2_C]^1.8+^:1.8e^–^	2D	2.9	[38]
Gd_2_C	2D	3.33 a	[46]
[Hf_2_S]^2+^:2e^−^	2D	3.6	[47]
Rb_3_O	3D	–	[42]

a Calculated value.

Based on their formation conditions, electrides can also be classified into ambient-pressure electrides and high-pressure electride [48,49]. The latter can only be discovered under high-pressure conditions, such as the transparent sodium electride [50] and a recent iron-light-element alloy, *hcp*-Fe(H, C, or O)_x_ (*x* < 1) [51].

### 2.3 Intermetallic electrides

Although numerous inorganic ionic electrides have been extensively studied, their limited stability in air or aqueous environments has restricted their practical applications. Intermetallic compounds, composed of two or more metallic or semimetallic elements arranged in a well-defined crystalline lattice, offer a promising alternative. It wasn't until 2016 that Y_5_Si_3_ was identified as the first intermetallic electride, showing remarkable stability in both air and water. In intermetallic electrides, anionic electrons are localized in crystallographic voids, and their presence is confirmed by the observation of an interstitial band crossing the Fermi level. Following this discovery, more intermetallic compounds with well-defined electride properties have been reported, including honeycomb-lattice compounds (CaAlSi, SrAlSi, BaAlSi) [52], Mn_5_Si_3_-type compounds (Y_5_Si_3_, Yb_5_Sb_3_, Sr_5_P_3_) [11,34], RTX compounds (LaScSi, LaCu_0.67_Si_1.33_, LaRuSi, where R denotes a rare-earth element, T a transition metal, and X a p-block aspect) [19,53,54], Sr_2_Bi-type compounds [13], Y_3_Pd_2_ [21], etc. Their exceptional stability is primarily attributed to significant hybridization and strong interactions between the electron anions and the surrounding rare-earth atoms, which shield and stabilize the anionic electrons. This unique bonding environment underpins the robustness of intermetallic electrides, even under ambient conditions, setting them apart from many conventional ionic electrides.

However, identifying and predicting intermetallic electrides pose certain challenges, as these compounds often deviate from traditional bonding models, such as the octet rule. For example, Y_5_Si_3_ and LaScSi can be formally written as [Y^3+^]_5_[Si^4–^]_3_:3e^–^ and [La^3+^][Sc^3+^][Si^4–^]:2e^–^, respectively, though the number of anionic electrons per formula unit is significantly <3 and 2. In LaRuSi, the anionic electrons arise due to the 14-electron rule [55]. Recent advances in high-throughput computational screening and machine learning have provided a feasible way to discover electride compounds through databases like the Materials Project Database and the Inorganic Crystal Structure Database (ICSD) [46,[56], [57], [58], [59], [60]]. This combination of experimental breakthroughs and theoretical innovations positions intermetallic electrides as promising candidates for future technological applications. For instance, Wang and co-workers pioneered the use of machine learning methods to efficiently screen electrides, and *A*_2_*BC*_2_-type electrides have been predicted [56], where highly symmetrical octahedral voids are surrounded by A and B atoms. Despite the similar electronegativity between A and B atoms, the greater polarizability of the A atom causes its valence electrons to migrate to interstitial sites under the influence of electrostatic fields within the crystal lattice. Meanwhile, valence electrons from the B atoms transfer to the C atoms to maintain charge balance. Y_2_LiSi_2_ is a representative *A*_2_*BC*_2_-type electride and the first known electron-deficient electride, which demonstrates high chemical stability and catalytic activity.

## 3 Electride-based materials for catalytic reaction

### 3.1 Overview

Catalysts are essential for advancing research in chemical reactions and industrial processes, as they are able to lower activation energy, reduce resource consumption, increase reaction rates, and enable high conversion efficiency with superior selectivity for target products. Electrides, with their distinct properties of low work function and high electron density, emerge as exceptional candidates for catalytic applications. Their anionic electrons, which are nucleus-free electrons, introduce a new chemical concept for catalyst design. These materials play a pivotal role in heterogeneous catalysis, either by directly using electrides containing active catalytic metal centers or by loading active metals onto electride surfaces. The nature of the active centers determines the types of reactions and functionalities that these electrides can catalyze. Compared to conventional catalysts, electride-based catalysts facilitate more efficient electron transfer during catalytic processes, offering superior performance in various reactions. This advantage is evident in their ability to operate under milder conditions while more effectively activating reactants.

To date, electrides have primarily been explored in heterogeneous gas-phase synthesis and organic synthesis. Notably, electride-based catalysts have shown an order of magnitude higher catalytic activity for ammonia synthesis, presenting a promising approach for mild-condition ammonia production [15,61]. While the application of electrides in electrocatalysis and photocatalysis is still in its early stages, the potential for future development in these areas is substantial. In this session, we summarize recent advancements in electride-based catalysts, focusing on their application in typical heterogeneous catalytic reactions, such as ammonia synthesis, CO*_x_* activation, and organic reactions, as well as their emerging roles in electrocatalytic and photocatalytic processes.

### 3.2 Ammonia synthesis

Ammonia synthesis is essential in global modernization and the food industry, serving as a fundamental component in producing fertilizers that support agricultural productivity worldwide [62]. The conventional industrial NH_3_ synthesis method, known as the Haber-Bosch process [63], is typically carried out at high pressures of 10∼30 MPa and temperatures of 400∼500 °C, yielding approximately 20% NH_3_ concentration in the effluent, presenting significant energy and resource challenges. Currently, on-site and small-scale green ammonia production processes have received growing attention for developing catalysts under milder conditions, where N_2_ activation is considered a critical step in the related mechanism. A landmark development in this field occurred in 2012 when Hosono et al. reported Ru-loaded C12A7:*e*^−^ electride catalyst exhibiting an exceptional property for NH_3_ synthesis under mild reaction conditions (temperature below 400 °C and pressure below 5 MPa) [18]. It is highlighted that the presence of hydride ions (*H*^−^) and anionic electrons within the support materials is essential for achieving outstanding catalytic performance in the low-temperature synthesis of NH_3_, and electride materials can confer these advantages [64]. Since then, various electride-based materials have been investigated, including those supporting transition metals [65] and intermetallic electrides such as LaCoSi and LaRuSi, which host active centers that do not require additional loading [12,66,67]. The design concepts, catalytic performance and related mechanisms of electrides in ammonia synthesis have been reviewed and summarized elsewhere [15,68], which will not be discussed in detail herein.

### 3.3 CO_x_ activation

In addition to ammonia synthesis in a reductive environment, electride-based catalysts have demonstrated potential in catalyzing oxidative reactions. It has been reported that C12A7:*e*^−^ remains stable up to 300 °C in the air, but oxygen/OH^–^ ions could be substituted for the encapsulated anionic electrons in an oxygen-rich atmosphere above 400 °C [69]. This allowed C12A7:*e*^−^ to function effectively as an electron-donor catalyst for oxidative reactions below 300 °C. Compared to Ru supported on conventional oxides, Ru nanoparticles (NPs) on C12A7:*e*^−^ exhibited superior oxidation resistance, attributable to the unique electronic property of C12A7:*e*^−^ [70]. X-ray absorption fine structure (XAFS) analysis has revealed that approximately 60% of 10 nm diameter Ru nanoparticles remain unoxidized even at temperatures up to 330 °C in the presence of O_2_ gas. These features endow Ru/C12A7:*e*^−^ with unique catalytic oxidative properties. M. J. Sharif et al. have shown that Ru/C12A7:*e*^−^ exhibited stable and highly active performance in catalyzing CO oxidation reactions [71].

**Fig. 3:**
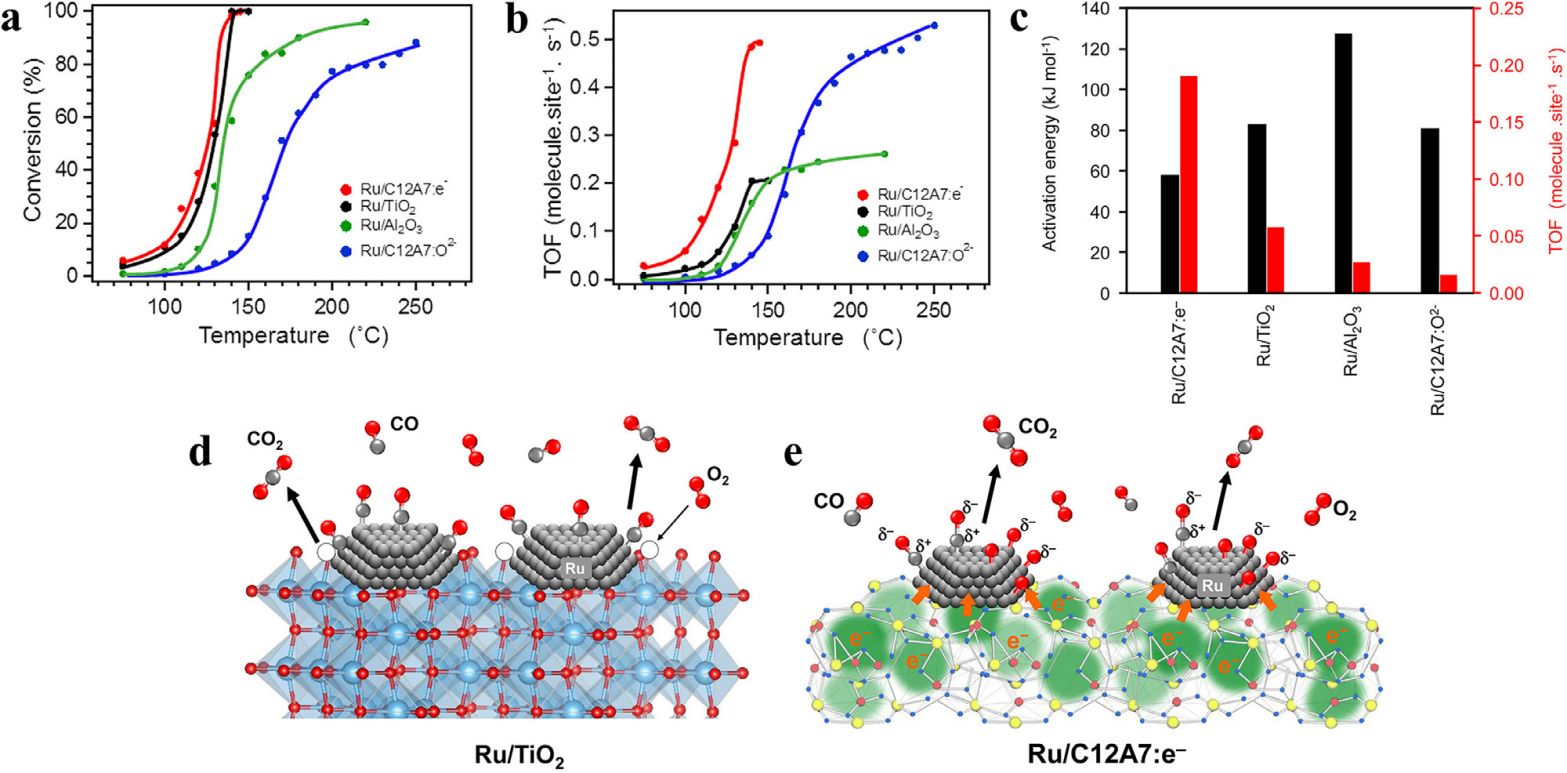
(a) Conversion ratio and (b) TOF as a function of reaction temperature for CO oxidation over various Ru catalysts. (c) TOFs and activation energies of the tested Ru catalysts. Reaction pathways for CO oxidation on (d) Ru/TiO_2_ and (e) Ru/C12A7:*e*^−^. Adapted with permission from ref [71]. Copyright 2015 American Chemical Society.

The onset temperature for the CO oxidation reaction over Ru/C12A7:*e*^−^ (∼70 °C) is much lower than that for Ru/C12A7:O^2−^ (∼100 °C), highlighting the catalytic functionality of the anionic electrons (Fig. 3a). Despite a much smaller surface area (∼1 m^2^·g^–1^) than that of the bench-mark Ru/TiO_2_ (54 m^2^·g^–1^), Ru/C12A7:*e*^−^ exhibits comparable apparent catalytic activity (Fig. 3a) [71], demonstrating that the turnover frequency (TOF) of Ru/C12A7:*e*^−^ is much greater. Ru/C12A7:*e*^−^ achieves 100% CO conversion at approximately 140 °C with a TOF of about 0.5 *s*^−1^ (0.19 *s*^−1^ at 120 °C), which is the highest value among various types of catalysts (Fig. 3b). The apparent activation energy of Ru/C12A7:*e*^−^ is also significantly lower than other catalysts (Fig. 3c).

The excellent catalytic performance of Ru/C12A7:*e*^−^ was mainly ascribed to the strong electron-donation capability of the electride substrate, which was shown to be distinct from the mechanisms of conventional catalysts. In conventional oxides, the prevailing understanding is that the surface lattice oxygen could facilitate the activation and oxidation of CO via the Mars-van Krevelen (MvK) mechanism [72]. The Mars-van Krevelen mechanism (MvK) is a well-established model for describing the role of lattice oxygen in catalysis. In the first step, reactants interact with the lattice oxygen on the catalyst surface, leading to their oxidation into products, while oxygen vacancies are generated in the catalyst. The second step involves the adsorption and dissociation of oxygen to replenish the oxygen deficiency, enabling reoxidation and regeneration of the catalyst. Within the framework of the MvK mechanism, the formation and conversion of surface oxygen vacancies are regarded as key steps. For instance, in comparison to the nonreducible Al_2_O_3_ (formation of oxygen vacancy is difficult), the reducible TiO_2_ exhibited a much higher catalytic activity (Fig. 3a), in which the lattice oxygen near the interface of metal-support participated in the reaction and then was regenerated by O_2_ adsorption (Fig. 3d) [72]. The CO oxidation process on Ru/C12A7, on the other hand, followed a distinct pathway from the MvK mechanism due to the significant energy barrier for removing framework oxygens even at temperatures exceeding 700 °C [10]. As shown in Fig. 3e, upon adsorption of O_2_ molecules and CO molecules on the Ru surface, O_2_ quickly dissociates into oxygen adatoms. Typically, the CO–O bond formation was reckoned to be the rate-determining step (RDS) in CO oxidation over Ru catalysts [73]. Thanks to the C12A7 electride, the energy barrier for this crucial step is significantly lowered. Anionic electrons are transferred from C12A7:*e*^−^ to Ru, facilitating a smooth charge transfer from the Ru d-orbital to the 2π* antibonding orbital of CO. Consequently, this process weakens the C–O bond, leading to the formation of C^δ+^–O^δ−^ species. Furthermore, the electron interaction between Ru and C12A7:*e*^−^ facilitates the production of negatively charged O adatoms (O*^δ^*^−^ species). Therefore, the reaction between CO and O results in CO_2_ formation, boosting catalytic performance and reducing the activation energy effectively.

**Fig. 4:**
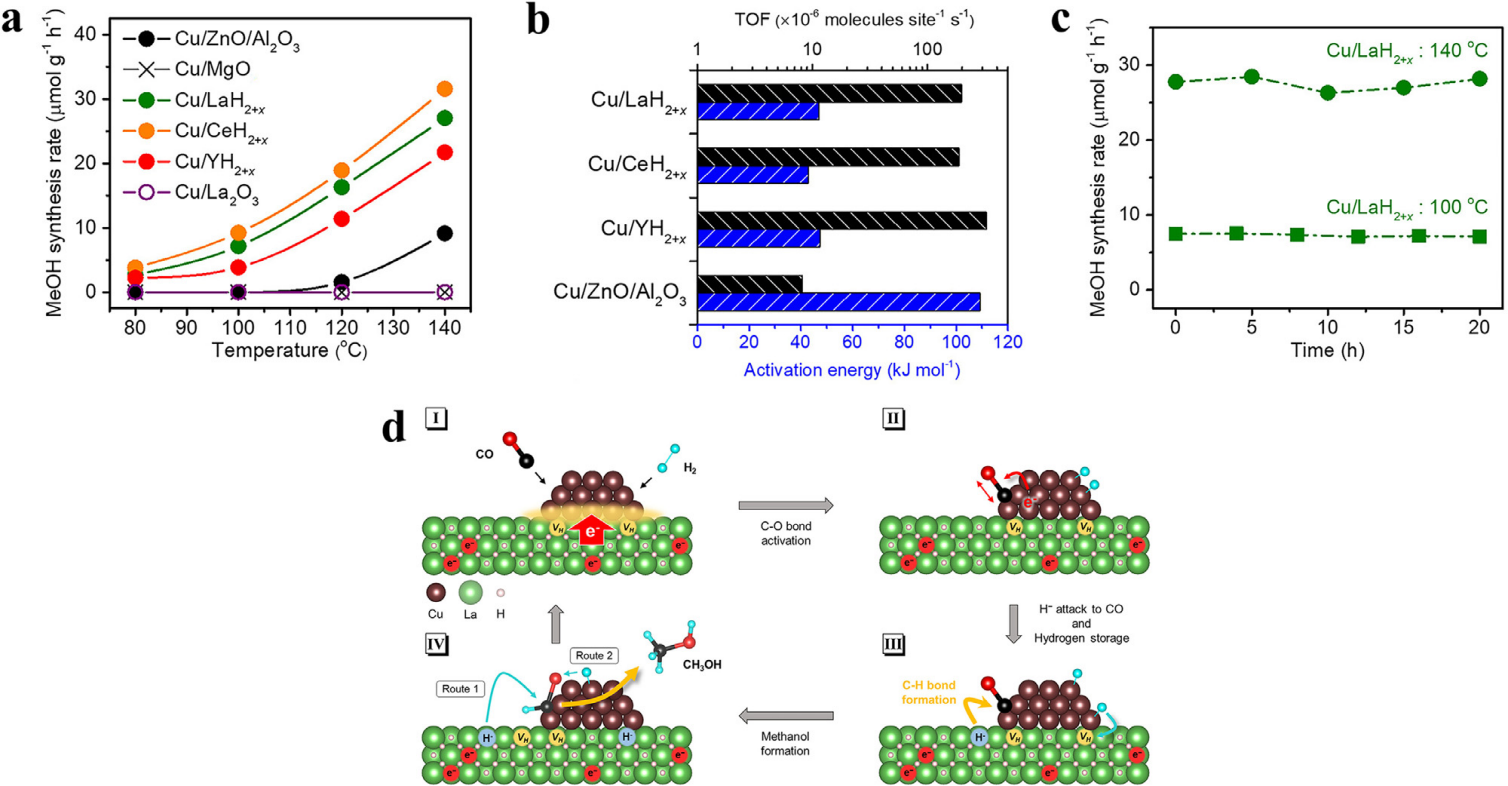
(a) Methanol synthesis rate over various Cu-loaded catalysts as a function of reaction temperature. (b) TOFs at 140 °C (black bars) and apparent activation energies (blue bars). (c) Time course at 100 and 140 °C over the Cu/LaH_2+_*_x_* catalyst. (d) Proposed reaction mechanism for methanol synthesis over Cu/LaH_2+_*_x_*. Adapted with permission from ref [20]. Copyright 2022 American Chemical Society.

The industrial process for methanol production through CO hydrogenation using H_2_ traditionally involves employing the Cu/ZnO/Al_2_O_3_ heterogeneous catalyst under high pressure and temperature conditions. To develop effective catalysts that can facilitate methanol synthesis under milder conditions, Sugiyama et al. have prepared Cu-loaded REH_2+_*_x_* electride materials (RE represents a rare-earth element) [20]. The Cu (5 wt%)/REH_2+_*_x_* catalysts demonstrated activity at 80 °C, whereas Cu (5 wt%)/La_2_O_3_ and Cu (5 wt%)/MgO exhibited no catalytic effect below 140 °C and 0.1 MPa (Fig. 4a). Compared to the industrial benchmark Cu (58 wt%)/ZnO/Al_2_O_3_ catalyst, the Cu/REH_2+_*_x_* catalysts also displayed significantly higher catalytic activities, as evidenced by the much smaller activation energy ranging from 43 to 47 kJ·mol^–1^ and a 20-fold higher TOF at 140 °C (Fig. 4b). Furthermore, Cu/LaH_2+_*_x_* catalyst showed long-term stability in a reducing reaction atmosphere (Fig. 4c).

It was further demonstrated that methanol synthesis using Cu/LaH_2+_*_x_* proceeded through a lattice H^–^ ion-mediated MvK mechanism. Density functional theory (DFT) calculations indicated that CO adsorption on Cu/LaH_3_ was more stable than on the Cu(111) metal surface. The formation of the first C–H bond (CO + *H* → HCO) was identified as the possible RDS for methanol synthesis over Cu-based catalysts, with the activation energy on the Cu/LaH_3_ being 52 kJ·mol^–1^, significantly lower than the value on the Cu(111) surface (99 kJ·mol^–1^) and the previously reported data on the Cu(211) surface (85–110 kJ·mol^–1^).

The proposed mechanism for methanol synthesis (Fig. 4d) involves the adsorption of CO and H_2_ molecules on negatively charged Cu, facilitated by electron donation from LaH_2+_*_x_* support (step I). Subsequently, the C–O bond is weakened due to the electron donating from Cu to the 2π*-antibonding orbitals of CO (step II). Following this, H^–^ ions from the hydride supports attack the activated CO molecule to facilitate the formation of the C—H bond. Hydrogen adatoms on Cu are then migrated and stored in the hydrogen vacancies of the hydride supports (step III), ultimately leading to the formation of methanol through further hydrogenation steps (step IV). Despite these advancements, the Cu-loaded REH_2+_*_x_* electride catalyst would suffer from a severe chemical stability issue in methanol synthesis from the more favorable CO_2_ and H_2_. The formation of byproduct water would deactivate the catalyst under synthesis conditions, highlighting the need to develop hydride-containing compounds with higher chemical stability.

CO_2_ activation has been an emerging topic in converting to other valuable chemicals to achieve carbon neutrality [74,75]. Traditional solid-state bases and transition metal surfaces are able to absorb and activate CO_2_, respectively. However, the activation process may occur under harsh experimental conditions due to the non-polar nature and two C = O double bonds in CO_2_. Toda et al. found that the high anionic electron concentration of C12A7:*e*^−^ in the cages could significantly enhance the absorption and dissociation of CO_2_ molecule on the C12A7:*e*^−^ surface into CO and O atoms at room temperature [76]. The anionic electrons occupying the cage structures on the C12A7:*e*^−^ surface served as adsorption sites for capturing CO_2_ and CO molecules.

**Fig. 5:**
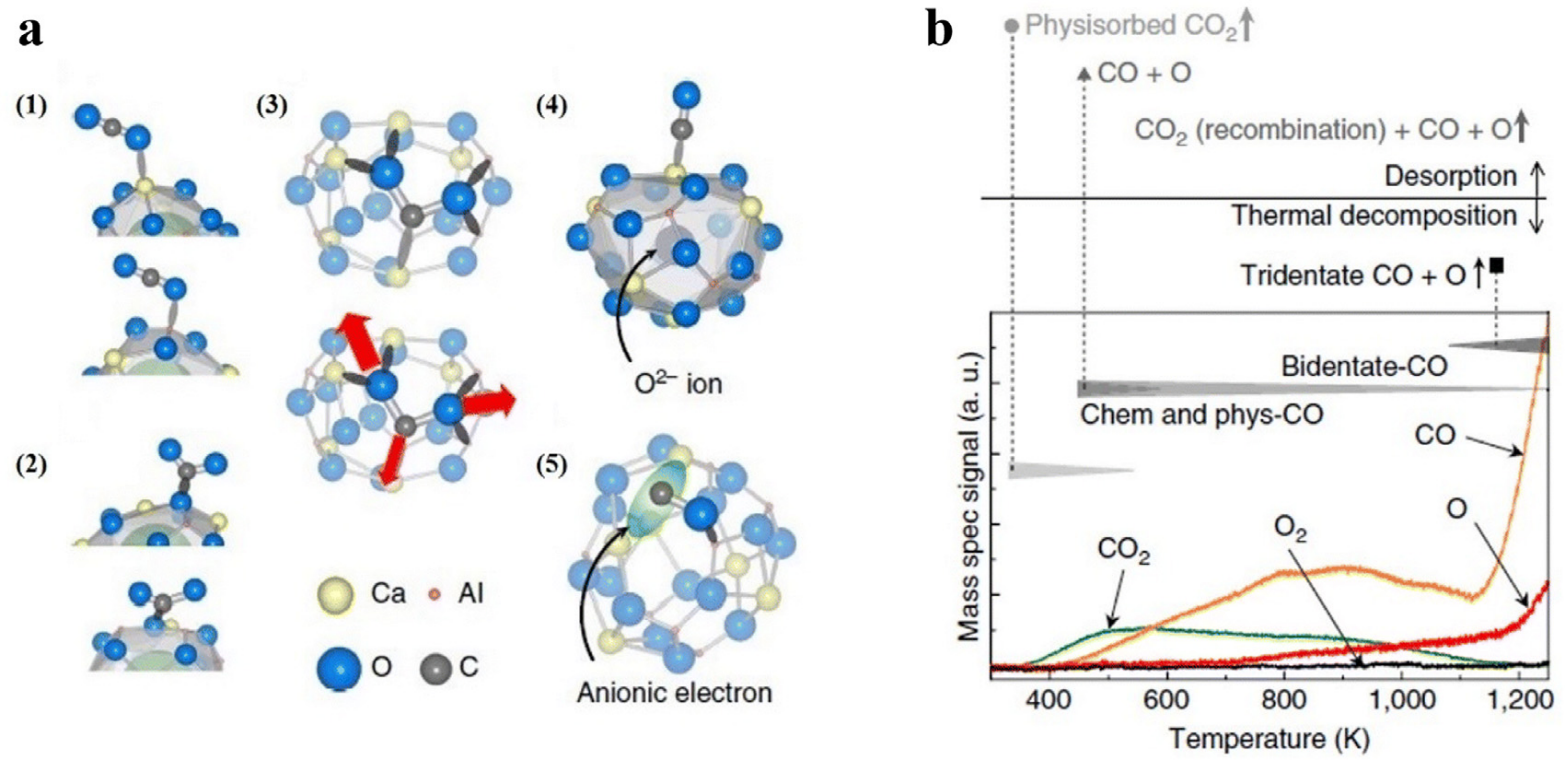
(a) Geometrical configurations of a CO_2_ molecule physisorbed on C12A7:*e*^−^ surface. (b) TPD curves of all desorption products from the C12A7:*e*^−^ surface after 2 L of exposure to CO_2_ at RT with the proposed CO_2_ desorption and decomposition sequence. The intensities of the curves were calibrated by sensitivity factors. The heating rate was 0.5 K *s*^−1^. Adapted with permission from ref [76]. Copyright 2013 Springer Nature.

As shown in Fig. 5a, various molecular adsorption configurations can occur on the corrugated surface of C12A7:*e*^−^, including (1) physisorbed CO_2_, (2) bent CO_2_*^δ^*^−^, (3) tridentate CO_2_ and (4, 5) dissociated CO + *O*. The temperature-programmed desorption (TPD) spectrum in Fig. 5b indicates that the physically adsorbed CO_2_ and CO molecules began to desorb at 350 and 400 K, respectively. Limited recombination of CO and O leads to additional CO_2_ desorption above 400 K. In comparison, O atom desorption restricts this recombination above 700 K. At temperatures exceeding 1100 K, both total physically adsorbed CO_2_ and most CO molecules are released from the surface. In addition, the deep-trapped CO_2_ in the tridentate configuration undergoes decomposition, leading to a rapid increase in the desorption of O and CO. The remarkable feature of C12A7:*e*^−^ as a reducing agent lies in its ability to activate and split CO_2_ without the need for harsh reaction conditions or reductive species, making it a promising candidate.

### 3.4 Organic reactions

Organic-ligand-based electrides are usually thermally unstable and extremely sensitive to moisture and air. However, Lu et al. recently synthesized a room-temperature stable organic electride for benzene/pyridine couplings and solvent-free Birch reduction [77]. The electride is capable of *K*^+^ or Li^+^ reduction by introducing a Lewis base ligand [78]. Inorganic electrides, such as C12A7:*e*^−^, Ca_2_N:*e*^−^, have been widely used as reducing reagents in pinacol coupling, Birch reduction or other reactions, which are summarized in Table 2. In these reactions, the electride framework decomposes in solution to release the electrons, which are immediately transferred to reactant molecules and thus promote reactions. It is noted that electrides in these reactions can only be used once due to the consumption of the trapped electrons. To achieve the recycling of the anionic electrons, many inorganic electrides have been employed as heterogeneous catalysts, including transition-metal-loaded electrides or transition-metal-containing intermetallic electrides, for various organic reactions. The results are summarized in Table 3.

**Table 2:** **Summary of chemical reactions using electrides as reducing agents**.

Electride	Reaction equation	Ref.
C12A7:e^−^		[17]
		[79]

Ca_2_N:e^−^		[80]
		[81]
		[82]
		[83]
		[84]

*K*^+^(LiHMDS):*e*^−^		[77]
		[77]

Ag-Ca_2_N:e^−^		[85]

**Table 3:** **Summary of organic reactions using electride as heterogeneous catalysts**.

Electride	Reaction equation	Ref.
Ru-Fe/C12A7:e^−^		[86]

LaCu_0.67_Si_1.33_		[19,87]
		[19]
		[88]
		[88]

Y_3_Pd_2_		[21]

Pd/Gr/C12A7:e^−^		[89]
		[89]

Cu NPs/Ca_2_N:e^−^		[90]

#### 3.4.1 Selective hydrogenation reaction

**Fig. 6:**
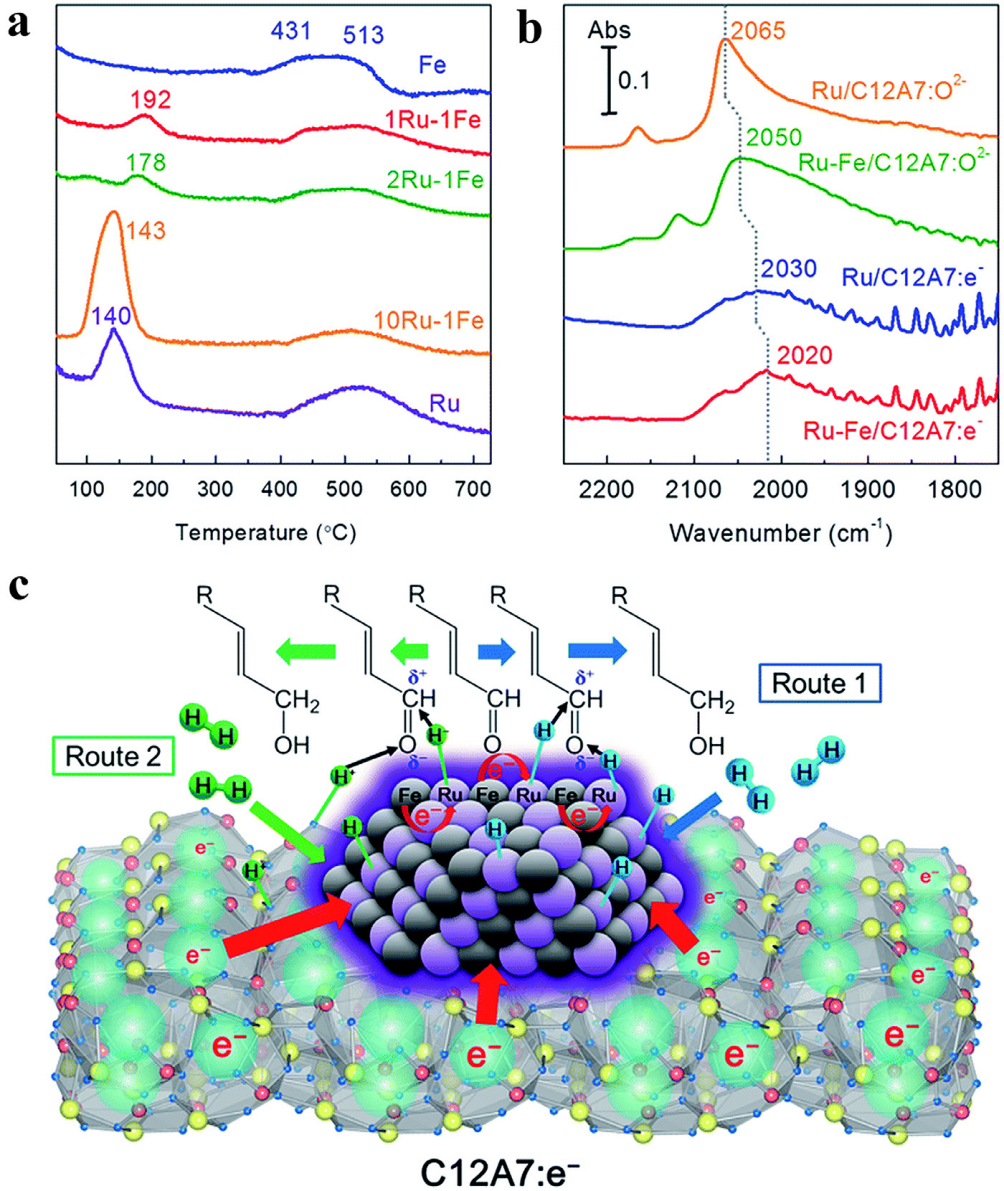
(a) H_2_-TPR profiles for several bimetallic Ru–Fe/C12A7:*e*^−^ catalysts with various Ru/Fe weight ratios: 2 wt% Fe/C12A7:*e*^−^, 1 wt% Ru–1 wt% Fe/C12A7:*e*^−^, 2 wt% Ru–1 wt% Fe/C12A7:*e*^−^, 10 wt% Ru–1 wt% Fe/C12A7:*e*^−^, and 2 wt% Ru/C12A7:*e*^−^. (b) Different DRIFTS spectra for adsorption of CO onto Ru/C12A7:O^2−^, Ru–Fe/C12A7:O^2−^, Ru/C12A7:*e*^−^, and Ru–Fe/C12A7:*e*^−^ at −170 °C under 5 kPa of CO. (c) Possible pathway for chemoselective hydrogenation of α,β-unsaturated aldehydes over Ru–Fe/C12A7:*e*^−^. Adapted with permission from ref [86]. Copyright The Royal Society of Chemistry 2016.

Catalytic hydrogenation is an essential and fundamental organic reaction in synthesizing natural products, pharmaceutical and pesticide molecules. Traditional catalysts generally involve transition metal catalysts such as Ni, Fe, Co, Pd, Ru, Pt, etc. The electron-rich nature of C12A7:*e*^−^ support facilitates the modification of the electronic structure of the loaded active metals, leading to changes in the configuration and strength of the adsorbed reactants. This modification is crucial for enhancing the selectivity and activity of metal-loaded catalysts. Combined with a Ru-Fe nanoalloy, C12A7:*e*^−^ has been shown to selectively catalyze the hydrogenation of α and β-unsaturated aldehydes into unsaturated alcohols [86]. Characterization using XRD and SEM revealed the formation of well-covered hexagonal Ru-Fe nanoparticles, each with a diameter of 15 nm, on the surface of C12A7:*e*^−^. In the H_2_-TPD measurements (Fig. 6a), as the Fe content in the Ru-Fe bimetallic catalyst increases, the hydrogen desorption peak over Ru sites shifted from 140 °C to 192 °C. Meanwhile, adding Fe caused a significant red shift in the Ru^0^-CO peak signal (2020 cm^−1^) in Diffuse reflectance infrared Fourier transform spectroscopy (DRIFTS, Fig. 6b), demonstrating the electronic interaction between Ru and Fe species. This bimetallic catalyst of Ru-Fe/C12A7:*e*^−^ exhibited extraordinarily high activity and selectivity in the hydrogenation of cinnamaldehyde, reaching 96.2% conversion with 96.7% selectivity for cinnamyl alcohol and could remain unchanged after eight cycles. For comparison, both Ru-Fe/C12A7:O^2–^ and Ru-Fe/Al_2_O_3_ without electron donor support showed poor selectivity, while the monometallic Ru or Fe-loaded C12A7:*e*^−^ gave inferior catalytic activity, and even their mixture could hardly enhance activity. These results indicate that the Ru-Fe bimetallic nanoparticles have a unique synergistic effect that improves the activity and the anionic electrons in C12A7:*e*^−^ are crucial for improving the selectivity. By controlling the anionic electron density, the selectivity of Ru-Fe/C12A7:*e*^−^ could be further tuned. Moreover, the Ru-Fe/C12A7:e^–^ was also proved effective in the hydrogenation of other unsaturated aldehydes with various functional groups, achieving high conversion and selectivity toward the corresponding unsaturated alcohols.

The mechanism of the selective hydrogenation of α, β-unsaturated aldehydes over Ru-Fe/C12A7:*e*^−^ is depicted in Fig. 6c. The electron transfer from C12A7:*e*^−^ to the Ru-Fe bimetallic nanoparticles reduces the interactions between the metal surface and the C **=** C bond, promoting a vertical adsorption configuration via the C **=** O bond. Electropositive Fe sites on the surface of Ru-Fe particles also function as electrophilic sites, binding to the C **=** O bond with the lone pair electrons of the O atom, thereby weakening the C **=** O bond. Consequently, the hydrogenation of the C **=** O bond is favored over the C **=** C bond. There are two potential pathways for H_2_ activation: (Route 1) homolytic cleavage and (Route 2) heterolytic dissociation (H_2_ → *H*^+^ + *H*^–^). H adatoms, produced by homolytic cleavage of H_2_, can stochastically react with both C **=** C and C **=** O bonds. Still, on Ru-Fe/C12A7:*e*^−^, the selective hydrogenation of the C **=** O bond is favored due to the specific adsorption configuration of aldehydes. In the case of heterolytic dissociation, H^–^ and *H*^+^ species are expected to form on the electron-rich metal sites and the framework oxygen sites of C12A7:e^–^, respectively, favoring the selective hydrogenation of polar functional groups C^δ+^ **=** O^δ−^ while retaining the C **=** C bonds.

**Fig. 7:**
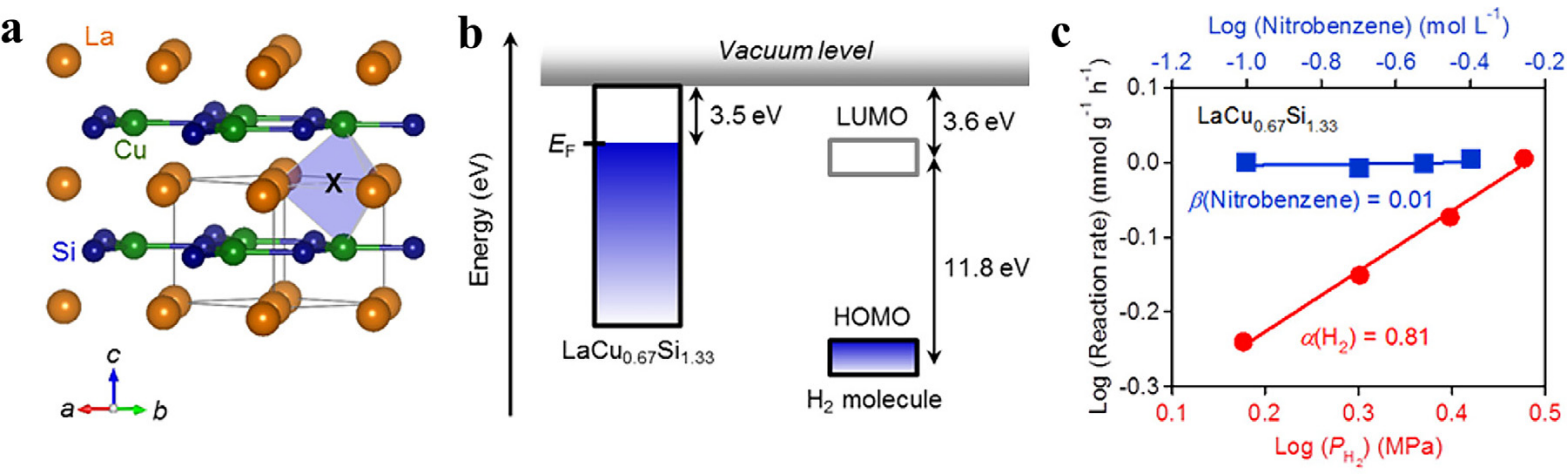
(a) Crystal structure of the stoichiometric LaCuSi. The La, Cu and Si atoms are depicted as orange, green and blue balls, respectively. The La_3_Cu_2_ cage is emphasized using a light-blue polyhedron. (b) Comparison of the Fermi level of LaCu_0.67_Si_1.33_ and the energy levels of H_2_. Here, LUMO denotes the lowest unoccupied molecular orbital, and HOMO denotes the highest occupied molecular orbital. (c) Dependence of reaction rate on nitrobenzene concentration and hydrogen pressure over the LaCu_0.67_Si_1.33_ catalyst. Adapted with permission from ref [19]. Copyright 2017 American Chemical Society.

Ye et al. have demonstrated that the intermetallic electride LaCu_0.67_Si_1.33_, featuring active lattice copper sites, exhibited high catalytic performance for the hydrogenation of nitroarenes, aldehydes, and ketones [19]. LaCu_0.67_Si_1.33_ crystallizes in a hexagonal structure, where Cu and Si atoms form a 2D honeycomb network and the La layer is located between each Cu/Si honeycomb layer (Fig. 7a). The band of anionic electrons crosses the Fermi level and the related electrons distribute around the interstitial sites within the La_3_Cu_2_ cages. The low work function of LaCu_0.67_Si_1.33_ (3.5 eV) is expected to efficiently promote the dissociation of H_2_ by enhancing electron donation to the LUMO (3.6 eV) of H_2_ molecules (Fig. 7b). The hydrogenation of nitrobenzene using LaCu_0.67_Si_1.33_ catalyst showed complete reaction without by-products. The aniline yield reached 99% in 9 h with a TOF as high as 5084 *h*^−1^, while the catalytic activities of Cu metal and LaSi were almost negligible. Even in a solvent-free system, the catalytic hydrogenation of 50 mmol nitrobenzene could be achieved with a yield of 99% under optimal conditions. The kinetic reaction orders were derived to be α (H_2_) = 0.81 and β (Nitrobenzene) = 0.01 (Fig. 7c), indicating that hydrogen pressure exerted a more substantial influence on the reaction rate compared to the concentration of nitrobenzene. Combined with the kinetic isotope effect of r_H_/r_D_ = 3.8 (>1), it was concluded that the cleavage of hydrogen molecules was considered as the rate-determining step. Isotope H_2_−D_2_ exchange reaction combined with DFT calculation further proved that lattice Cu atoms were responsible for hydrogen dissociation. The HD formation rate over LaCu_0.67_Si_1.33_ is significantly higher than that of LaSi without Cu, indicating that Cu sites in LaCu_0.67_Si_1.33_ function as active sites for hydrogen activation. The most stable adsorption site for oxygen (O) on LaCu_0.67_Si_1.33_ was the hollow site (h-LLC) consisting of one copper (Cu) atom and two lanthanum (La) atoms. The preferential adsorption of nitro at h-LLC sites and the activation of hydrogen at Cu sites are beneficial in improving the selectivity of the target product.

To further improve the catalytic performance, LaCu_0.67_Si_1.33_ electride nanoparticles were prepared using the Ar/H_2_ arc flash evaporation method. These particles exhibited a high surface area (SBET = 65.0 m^2^ *g*^−1^) and a 60-fold increase in catalytic performance for nitrobenzene hydrogenation [87].

#### 3.4.2 Cross-coupling reaction

The Suzuki cross-coupling reaction is a crucial method for carbon-carbon bond formation, commonly catalyzed by palladium-based complexes. It involves cross-coupling aromatic or alkenyl boronic acids or boronic acid esters with chlorine, bromine, or iodo-aromatic hydrocarbons or olefins to produce biaryls. This reaction shows strong substrate adaptability and functional group tolerance, which has been applied in synthesizing many natural products and organic compounds [91,92]. However, the homogeneous Pd-complex catalyst poses challenges concerning separation and purification from the reaction system, as well as the utilization of expensive and possibly toxic ligands. Developing alternative heterogeneous Pd catalysts in nanoparticles or single atoms has been considered an ideal solution. Nevertheless, the stability, possible aggregation and/or leaching of Pd sites from the support surface should be carefully taken into account in terms of recycling. In this regard, Ye and Lu et al. proposed that Pd-containing intermetallic electride materials were efficient heterogeneous catalysts for cross-coupling reactions, exhibiting high catalytic activity and stability [21,93].

**Fig. 8:**
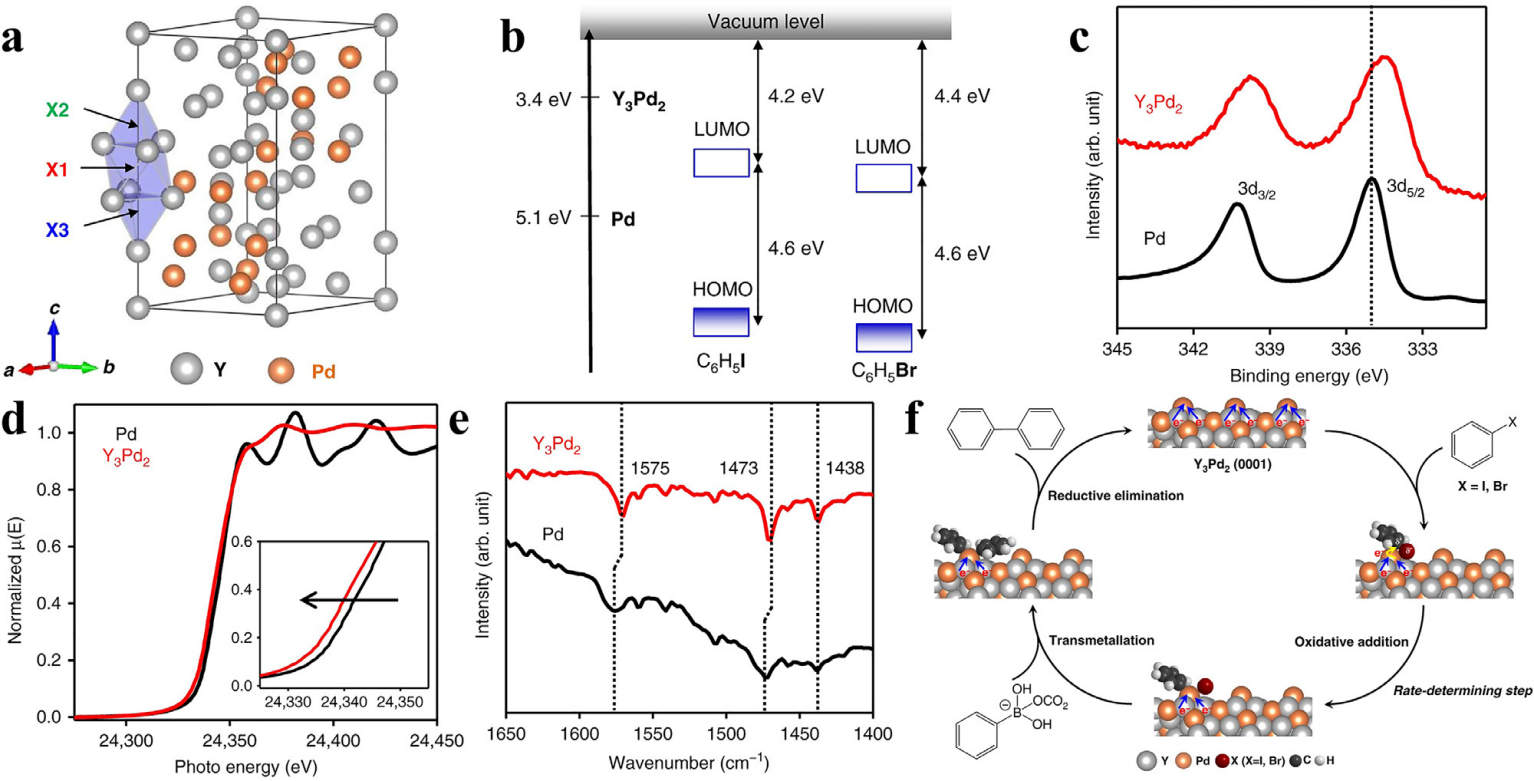
(a) Crystal structure of the stoichiometric Y_3_Pd_2_. Y and Pd atoms are depicted as gray and orange balls, respectively. X1, X2, and X3 sites are indicated using black solid arrows. (b) Comparison of the Fermi level for Y_3_Pd_2_ and the energy levels of aryl halides (iodobenzene and bromobenzene). (c) Pd 3d XPS spectra for the Pd metal and the Y_3_Pd_2_. (d) Pd K-edge XANES spectra for the Pd metal and the Y_3_Pd_2_. (e) DRIFTS spectra for the adsorption of iodobenzene onto Pd and Y_3_Pd_2_ at 25  °C, respectively. (f) Proposed reaction pathways for the Suzuki cross-coupling process over Y_3_Pd_2_. Adapted with permission from ref [21]. Copyright 2019 Springer Nature.

Y_3_Pd_2_ [21] obtained by the arc melting method adopts a trigonal structure, where Y atoms form Y_6_ octahedral and Y_4_ tetrahedral cages (Fig. 8a) with X1, X2, and X3 periodic cavities. According to DFT calculation, Y_3_Pd_2_ exhibits a metallic band structure, and the electronic energy bands near the Fermi level are related to the anionic electrons around the X1–3 sites. Further analysis showed that Y_3_Pd_2_ could be formally written as [Y_3_Pd_2_]^+^*e*^–^. Y_3_Pd_2_ has a much higher carrier density of 1 × 10^22^ cm^−3^ than traditional oxides and carbon materials. Its work function (3.4 eV) is much smaller than that for the Pd metal (≈ 5.1 eV), which would benefit aryl halide activation. High electron concentration in Y_3_Pd_2_ is critical to induce the smooth electron transfer from the catalyst to the LUMO of the organic halides: since the work function of Y_3_Pd_2_ is distinctly lower than the LUMO level, when the molecule adsorbed on Y_3_Pd_2_ Schottky barrier is formed at the interface, leading to suppression of electron transfer. Electron transfer, however, becomes possible by tunneling mechanism when the electron concentration is high like in metals [9]. Thus, electrons from Y_3_Pd_2_ can effectively transfer to the LUMOs of adsorbed aromatic halides (Fig. 8b), thereby lowering the activation energy necessary to break the C-X bond (*X* = *I* and Br). XPS (Fig. 8c) and K-edge XANES (Fig. 8d) measurements results indicated that Pd was electronegative in Y_3_Pd_2_; meanwhile, positively charged Y served as an electron-donating element, enhancing the electron density of Pd. DRIFTS analyses provided further insights into the electronic effect occurring during the activation process of the adsorbed molecules (Fig. 8e). Compared with Pd, the C-I stretching vibration of iodobenzene adsorbed on Y_3_Pd_2_ showed a redshift, indicating that the electron-donating from Y_3_Pd_2_ to iodobenzene weakened the C-I bond. Therefore, Y_3_Pd_2_ exhibited much better catalytic performance and suppressed specific activation energy for the Suzuki coupling of phenylboronic acid with iodobenzene than the pure Pd metal. Moreover, recycling experiments demonstrated the exceptional reusability of the Y_3_Pd_2_ catalyst without any loss of activity after 20 cycles, showcasing significantly greater stability compared to other reported Pd-based heterogeneous catalysts. The TOF of Y_3_Pd_2_ was an order of magnitude higher than those of benchmarked commercial heterogeneous Pd catalysts such as Pd/C, Pd/Al_2_O_3_, and Pd-Pb/CaCO_3_.

The potential reaction mechanism is depicted in Fig. 8f. The aryl halide molecules are adsorbed on the Y_3_Pd_2_ surface at the Y^δ+^-Pd^δ−^ sites through strong electrostatic interactions. The negatively charged Pd then acts as an electron donor to weaken the C-X bond and thus decrease the activation energy of the reaction. The electron transfer is a critical step in activating aryl halides, ultimately facilitating the efficient catalytic formation of the final coupling product through subsequent transmetalation and reductive elimination processes.

While displaying superior catalytic activity and stability, Y_3_Pd_2_ samples prepared using the arc-melting method exhibit a tiny surface area, and the utilization efficiency of Pd active sites is extremely low, limiting their potential for practical applications. To overcome this issue, Lu et al. have proposed a strategy to disperse the intermetallic ZrPd_3_ nanoparticles on the surface of the nanoporous ZrC, resulting in a low Pd content (1 wt%) as well as a large surface area of 340–450 m^2^ *g*^−1^ [93]. Similar to Y_3_Pd_2_, ZrPd_3_ also featured a negatively charged Pd and a low work function, which contributed to a low activation energy in Suzuki coupling reactions. Each aryl halide with different substituted functional groups could be converted to the corresponding coupled products in high yields (> 85%) under mild conditions. The ZrPd_3_ catalyst remained stable after 15 cycles and the TOF was comparable to Y_3_Pd_2_. The reaction mechanism, akin to Y_3_Pd_2_, also highlights the electron-transfer effect as one of its key features. DFT calculations indicated that when the reaction substrate approached the surface of the ZrPd_3_ cluster, electrons were transferred to bromobenzene, elongating and weakening the C-Br bonds. Due to the strong electron-donating ability and the negatively charged Pd active sites, ZrPd_3_ catalysts were also used for the selective hydrogenation of nitroaromatic hydrocarbons. The low Pd loading amount and the abundance of exposed Pd sites significantly improve the Pd-atom economy compared to previously reported catalysts.

Recently, a heterogeneous composite catalytic system (Pd/Gr/C12A7:*e*^−^) containing the active Pd metal, C12A7:*e*^−^ and conductive graphene (Gr) has been reported [89], where the C12A7:*e*^−^ was well encapsulated by the Pd/Gr multi-layers. Cross-coupling catalytic experiments demonstrated that the tested aryl halides with a range of functional groups would react with different types of boronic acids to produce corresponding coupled products in high yields, suggesting the general applicability of the Pd/Gr/C12A7:*e*^−^ catalyst with high selectivity and functional group tolerance. The TOF (1413.3 *h*^−1^) of Pd/Gr/C12A7:*e*^−^ even outperformed those of Y_3_Pd_2_, ZrPd_3_-ZrC and other commercial Pd-based catalysts. The good catalytic performance of Pd/Gr/C12A7:*e*^−^ was attributed to the efficient electron transfer from the inorganic C12A7:*e*^−^ electride to the graphene and the loaded Pd clusters. In the Gr/C12A7:*e*^−^ heterostructure, the low work function of the C12A7:*e*^−^ electride (Φ_WF_ = 2.4) enabled an electron donation from C12A7:*e*^−^ to Gr. Meanwhile, owing to the similar work functions of Gr (Φ_WF_ = 5.0 eV) and Pd (Φ_WF_ = 5.1 eV), electrons could quickly transfer between them. The X-ray absorption near edge structure (XANES) measurement confirmed that Pd species carried negative charges. Mechanistic studies demonstrated that the strong electron donation ability of the C12A7:*e*^−^ electride enabled negatively-charged Pd sites through a multistep electron transfer process to promote the activation of aryl halides, which was the rate-determining step for the investigated cross-coupling reactions. In addition, the Pd/Gr/C12A7:*e*^−^ catalyst was adequate for a range of C—C cross-coupling reactions, including Sonogashira, Stille, Hiyama and Heck coupling reactions.

#### 3.4.3 Other organic catalytic reactions

**Fig. 9:**
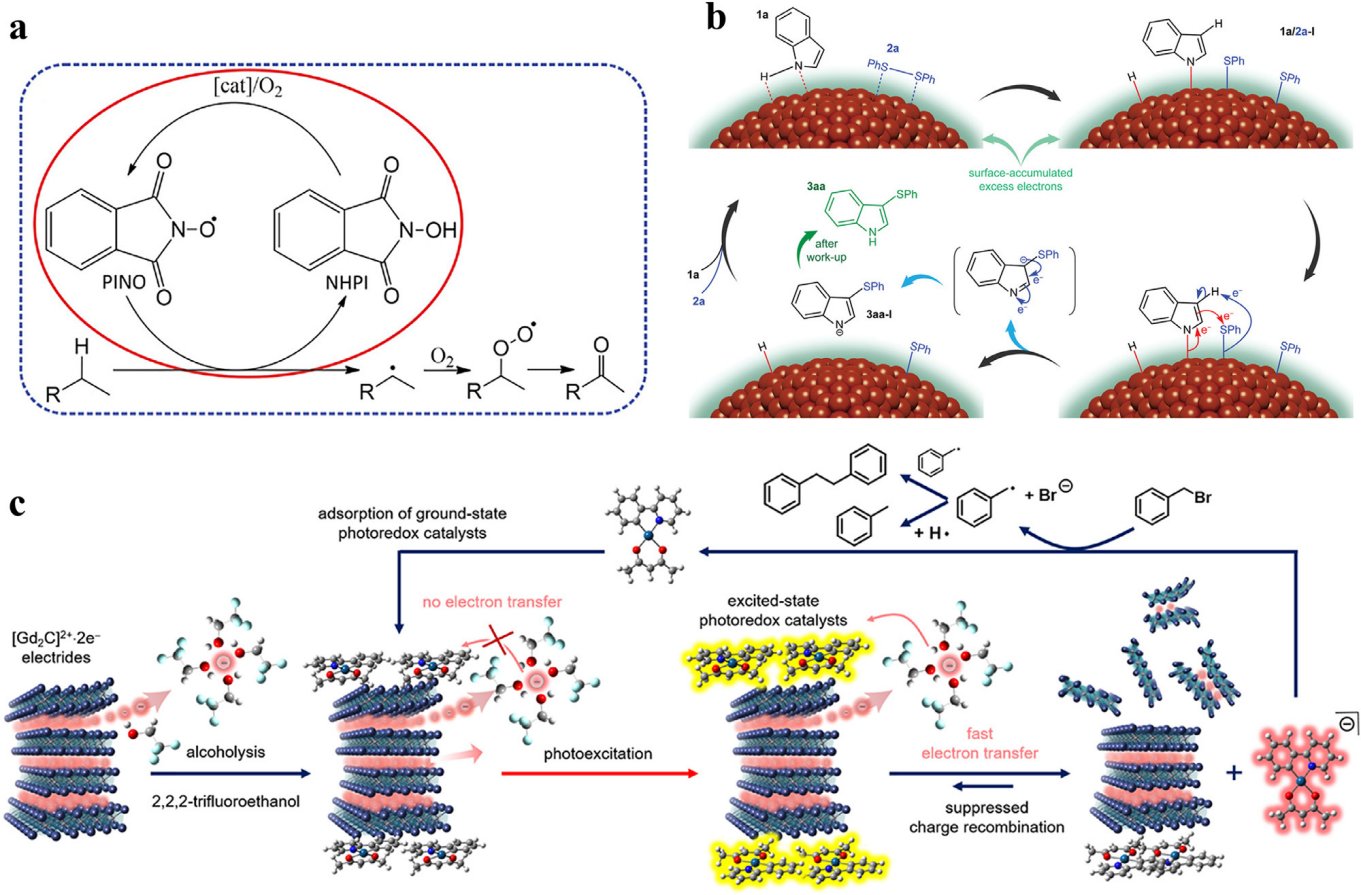
(a) Proposed mechanism of the C–H bond activation with an encircled theoretically investigated part. Adapted with permission from ref [88]. Copyright 2022 WILEY-VCH. (b) Proposed mechanism for the sulfenylation of indoles, using the depicted molecules 1a and 2a as examples. Adapted with permission from ref [90]. Copyright 2022 WILEY-VCH. (c) 2D electride (Gd_2_C:2e^−^) as persistent electron donors to promote photocatalytic redox reactions. Adapted with permission from ref [94]. Copyright 2021 American Chemical Society.

Kadam et al. used LaCu_0.67_Si_1.33_ for catalytic selective C—H oxidation and cycloaddition of CO_2_ with epoxides [88]. The Cu sites activated by anion electrons and the low work function enabled LaCu_0.67_Si_1.33_ to exhibit strong electron-donating ability during catalytic cycling, allowing the reaction to proceed under mild conditions. For this reason, LaCu_0.67_Si_1.33_ catalyst showed 98% conversion and 97% selectivity for C—H bond oxidation with a high TOF of 25,276 *h*^−1^ and 50–99% conversion with a TOF up to 800,000 *h*^−1^ for the cycloaddition of CO_2_ with epoxides into cyclic carbonates. Kinetic studies using electron paramagnetic resonance (EPR) and DFT calculations revealed that the catalytic selective C—H oxidation follows a radical mechanism. In this process, the highly electron-donating LaCu_0.67_Si_1.33_ can activate O_2_, aiding in the transformation of N-hydroxyphthalimide (NHPI) into the phthalimido-N-oxyl (PINO) radical (Fig. 9a). This activation step is vital for the subsequent selective H-abstraction from ethylbenzene and its further oxidation.

Despite the highly reactive nature of the 2D electride Ca_2_N:*e*^−^ in the reduction of organic molecules, Han et al. reported that the negatively charged surface of Cu NPs on Ca_2_N:*e*^−^ can catalyze the selective sulfenylation of indoles and azaindoles with diaryl disulfides [90]. The disparity in work functions between Cu NPs (approximately 4.5 eV) and Ca_2_N:*e*^−^ (approximately 2.6–3.5 eV) generates a significant interfacial electric potential, enabling charge transfer to Cu. Consequently, the work function of Cu NPs decreases (calculated at approximately 3.9 eV), and the surface hosts excess electrons, thereby preventing oxidation in the air. Meanwhile, Cu NPs become electronegative and possess an excess charge of 1.6 *e*^−^ per surface Cu atom. Such an electron-rich heterogeneous catalyst was subsequently employed in catalytic sulfenylation, resulting in yields of over 80% and displaying good tolerance to various substitutions. The proposed mechanism indicates that disulfide and N—H indole molecules undergo adsorption on the surface of the Cu nanoparticles. Cu interacts with S-S and N—H bonds to generate activated species. Due to its coordination with negatively charged Cu species, the indole group experiences an increased electron density, thereby promoting the sulfenylation reaction with the -SPh group on the surface of the Cu nanoparticles. Immediately, rapid deprotonation results in the formation of sulfenylated indole species, which are subsequently protonated in water to generate the final product (Fig. 9b).

Heo et al. have reported a novel photocatalytic system featuring Gd_2_C:2e^−^ and Pt(II) complexes as electron donors and photoredox mediators, respectively (Fig. 9c) [94]. The Gd_2_C:2e^−^ electride has a similar layered structure to Ca_2_N:*e*^−^, with its anion electrons more localized in the interlayer space. This robust electron localization likely contributes to higher intrinsic stability of Gd_2_C in 2,2,2-trifluoroethanol (TFE) solvent. According to luminescence titration and transient photoluminescence spectra, Pt(II) complexes are absorbed on the surface of Gd_2_C:2e^−^ and rapidly capture the electrons released from Gd_2_C:2e^−^ in TFE at a rate of 10^7^ *s*^−1^ upon photoexcitation. This process can be attributed to the more negative ionization potential of Gd_2_C:2e^−^ (−1.88 V vs. SCE) compared to the excited state reduction potential of Pt(II) complexes (∼0.26 V vs. SCE). This comparison implies the likelihood of a one-electron transfer from Gd_2_C:2e^−^ to the photoexcited state of Pt(II) complexes. The photoredox catalytic performance was evaluated by dehalogenation and homocoupling of benzyl halides. Under 365 nm irradiation and the optimized conditions, benzyl bromide was converted to bibenzyl (90%) and toluene (10%). Furthermore, the catalysis was tolerant of reactive halogen, ester and cyano groups, but less reactive with electron-withdrawing substituents.

### 3.5 Electrocatalyst and photocatalyst

In addition to heterogeneous thermal catalysis, electrides have also been investigated for electrocatalysis and photocatalysis. Li et al. proposed C12A7:*e*^−^ as an innovative cathode material in electrochemical reactions. Due to its metallic properties and low work function, the C12A7:*e*^−^ electrode exhibited high activity in the electrochemical conversion of p-(methylthio)-phenylboronic acid to p-(methylthio) phenol [95]. The cathodic reduction of oxygen at the C12A7:*e*^−^ electrode in acetonitrile, using [Bu_4_N]ClO_4_ as an electrolyte, produced a higher yield of superoxide ions (O_2_^•−^) compared to traditional Pt and glassy carbon (GC) electrodes. This leads to a remarkable 90% yield of p-(methylthio)phenol at a charge of 2.5 F·mol^−1^, surpassing the performance of Pt (65%) and GC (79%) electrodes. The research group also discovered the strong affinity of the C12A7:*e*^−^ electrode for CO_2_ and its ability to suppress CO_2_ reduction, promoting the efficient monocarboxylation of various olefins [96]. When galvanostatic electrolysis is conducted in a CO_2_ atmosphere, the C12A7:*e*^−^ electride yielded monocarboxylic products at moderate to high yields, ranging from 58% to 91%. These results surpass the yields obtained with commercial GC (32%−72%) and Pt (9%−83%) cathodes. By utilizing galvanostatic electrolysis and cyclic voltammetric analysis, it has been demonstrated that the olefin is initially reduced at the cathode to produce a radical anion, which promptly reacts with the adsorbed CO_2_ to selectively form the corresponding monocarboxylic products.

**Fig. 10:**
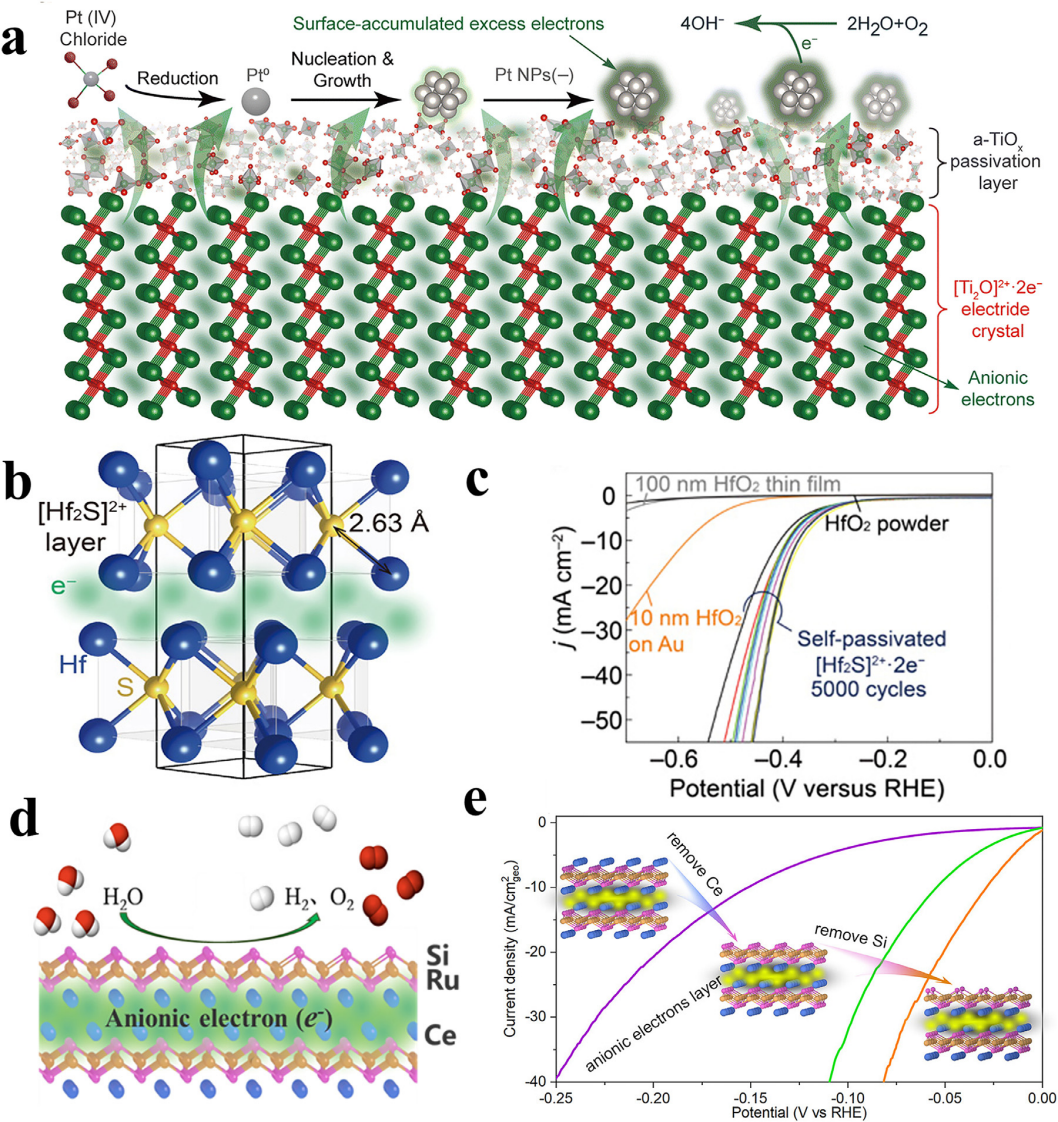
**Water-durable electride for electrocatalysis.** (a) Schematic illustration for preparing Pt NPs on the [Ti_2_O]^2+^:2e^−^. The green shadow areas indicate the anionic electrons. Adapted with permission from ref [99]. Copyright 2023 The Royal Society of Chemistry. (b) The lattice structure of Hf_2_S electride with the anionic electrons is schematically shown in the interlayer space. (c) Catalytic hydrogen evolution performance for the self-passivated Hf_2_S, HfO_2_ powder, and HfO_2_ thin films. (b) and (c) are adapted with permission from ref [47]. Copyright 2020 American Association for the Advancement of Science. (d) The lattice structure of the intermetallic electride CeRuSi and the schematic diagram of the water splitting process. (e) Catalytic hydrogen evolution performance of CeRuSi with its surface modified through chemical etching. (d) and (e) are adapted with permission from ref [22]. Copyright 2023 American Chemical Society.

Khan et al. have reported the utilization of nanosized Sn or Fe-doped C12A7:*e*^−^ composite material in conjunction with the reduced graphene oxide to enhance conductivity. This approach significantly heightened the long-lasting activity during electrochemical oxygen reduction reaction (ORR) in fuel cells [97,98]. The ORR current densities are higher than that of commercially available Pt/C electrode. Remarkably, Hua et al. have recently prepared Pt NPs supported by [Ti_2_O]:2e^−^ electride through an in-situ reduction of PtCl_4_ for electrocatalytic ORR (Fig. 10a) [99]. The [Ti_2_O]:2e^−^ electride generates a thin and conductive self-passivated TiO_2-x_ layer, providing good air and water stability. Pt NPs on the [Ti_2_O]:2e^−^ electride acquire a negative charge through metal-support interaction-induced charge transfer from [Ti_2_O]:2e^−^ to Pt. The excess electrons on the surface inhibit Pt-O formation in alkaline media. The electrocatalytic ORR activity of Pt-[Ti_2_O]:2e^−^ exhibits specific and mass activities that are 89 and 31 times higher, respectively, compared to commercial Pt/C. Specifically, it achieves a specific activity of 14.2 mA cm_pt_^−2^ and a mass activity of 3.1A mg_pt_^−1^ at 0.4 V. The catalyst also demonstrates durability, maintaining a high rate of 95% after 350 h of operation.

Kang et al. have reported the discovery of a transition metal-rich monochalcogenide electride [Hf_2_S]^2+^:2e^−^, which exhibits HER activity [47]. Hf_2_S:2e^−^ adopts an anti-TaS_2_–type crystal structure, with the anionic electrons residing in the 2D interlayer space between the Hf cationic layers (Fig. 10b). Different from most ionic electrides, Hf_2_S:2e^−^ displays robust stabilities against water and acid, positioning it as an ideal electrocatalyst. The amorphous Hf_2_S:2e^−^ layer on the surface reacts with oxygen, forming a protective outermost amorphous HfO_2_ layer with a thickness of approximately 10 nm, thus preserving its integrity. Electrocatalytic HER tests were conducted in a 0.5 M sulfuric acid solution. The self-passivated Hf_2_S:2e^−^ electride exhibited an overpotential of 355 mV at a current density of 10 mA cm^−2^ with a Tafel slope of 130 mV decade^−1^. For comparison, the HER tests were also performed on the 100-nm-HfO_2_ thin film and HfO_2_ powders. They showed nearly no HER activity (Fig. 10c). These results indicated that the excess electrons in the self-passivated electride could transfer through the thin HfO_2_ passivation layer under the applied electric field, contributing to sustained electrocatalytic HER performance. In addition to the experimental investigations, computational studies were also performed to evaluate the ORR and oxygen evolution reaction (OER) performance of other 2D electrides that formed heterojunctions with graphene. It was demonstrated that adjusting the work functions could effectively control the charge transfer process, and the transferable electrons could tune the d-orbital occupation of loaded transition metal single atoms, resulting in improved electrocatalytic performance [100].

Shen et al. recently demonstrated that the intermetallic electride LaRuSi is an effective electrocatalyst for alkaline HER, showing an overpotential of 72 mV at a current density of 10 mA·cm^−2^, closely approaching that of commercial Pt/C [54]. Cai et al. also developed a quasi-2D intermetallic electride CeRuSi (Fig. 10d) with significantly enhanced catalytic activity for HER in alkaline media [22]. With a surface area of only ∼1 m^2^·*g*^−1^, CeRuSi exhibits a very low overpotential of 28 mV at a current density of 10 mA·cm^−2^ as well as long-term stability in 1.0 M KOH. This makes it the best electride-based electrocatalyst for HER to date. The Tafel slope was calculated to be 24 mV·dec^−1^, indicating that the RDS is the Tafel step with significantly accelerated kinetics. The exceptional activity can be attributed to the unique features of electrides and the presence of coordinatively unsaturated Ru sites. Through selective chemical etching to remove the surface Ce and Si, the number of unsaturated Ru sites is increased and the active surface area is expanded, while simultaneously optimizing the H binding energy on the Ru catalytic active sites (Fig. 10e). For comparison, the HER activities of the isostructural CeScSi electride (the overpotential at 10 mA/cm^2^ and Tafel slope are 800 mV and 576 mV·dec^−1^, respectively) and the non-electride material LaRu_2_Si_2_ (the overpotential at 10 mA·cm^−2^ and Tafel slope are 460 mV and 283 mV·dec^−1^, respectively) were also found to be significantly lower than those of CeRuSi. These results reveal that both the Ru activation centers and the electride features are crucial for excellent HER performance.

In addition, recent studies have shown that C12A7:*e*^−^ nanoparticle prepared by the sol-gel method can be used as a promoter for P25 (10 wt% NiSe/TiO_2_) in the photocatalytic production of H_2_ from water splitting [101]. Prepared using a simple mechanical mixing method with the addition of 0.25 wt% C12A7:*e*^−^, the composite exhibits an optimal H_2_ evolution rate of up to 663 μmol·*h*^−1^·*g*^−1^, marking a substantial 43.9-fold increase compared to pure P25. The enhanced photocatalytic activity is attributed to the high electron-donating capability of C12A7:*e*^−^ and the built-in electric field formed from P25 to C12A7:*e*^−^ resulting from the variance in conduction band energy.

## 4 Summary and outlook

This paper reviews recent developments of electride-based catalysts, including the well-known C12A7:*e*^−^, other inorganic ionic electrides, intermetallic electrides, etc. Novel electride materials have demonstrated remarkable capabilities as excellent catalysts for a wide range of chemical reactions, including ammonia synthesis, CO*_x_* activation, organic reactions, as well as electrochemical and photochemical reactions. Notably, these materials exhibit outstanding stability, activity and selectivity in the corresponding catalytic applications. The superior catalytic performance is primarily attributed to the high electron densities and low work-function properties of electrides. These characteristics enable efficient electron transfer to the LUMOs of the adsorbed substrate molecules, facilitating the activation of resource small molecules such as N_2_, O_2_, H_2_, NH_3_, CO_2_, and CO, with significantly reduced activation energies. In most cases, the active transition metals are either embedded within the lattice as one component of the electrides or loaded as NPs on electride surfaces. In these cases, the catalytic activities and selectivity heavily depend on the negatively charged transition metal centers attributed to the electron donation from the anionic electrons in electrides. Overall, electrides have provided an inspiring research platform in catalysis. Several perspectives in the future development of electride materials in the field of catalysis include:

1. Enlargement of the specific surface area: So far, electrides have principally been synthesized by arc-melting or solid-state reaction methods. Typically, the resultant samples exhibit low-surface areas, which will significantly reduce the available active sites directly interacting with the reaction substrate, thereby limiting the number of surface-active sites. Therefore, developing synthetic strategies for nanoscale electrides that can uphold catalytic activity while preserving electron donation remains a challenging topic in the field of catalysis. Synthetic technologies, such as the hydrothermal method, the molten-salt method, the flash-evaporation method, and the deposition methods, can potentially minimize the particle size of electride materials.

2. Enhancement of catalytic active sites via surface engineering: Surface modification can not only alter the distribution and coordination environment of the active centers by removing inactive components or adding active components, but also induce significant changes in the local electronic and geometric structure of the interface, thereby affecting the activity and selectivity of the catalyst. In practical catalytic systems, the surface structure of catalysts can be influenced by various external factors, such as surface etching, heat treatment, addition of reducing agents, deposition coatings, and others. These factors can result in different catalytic performances compared to ideal, pristine catalytic systems that do not consider environmental influences. These technologies provide a high level of tunability, allowing researchers to customize the surface properties of catalysts to suit various catalytic applications, reactions, and operating conditions.

3. Development of new electride materials with stability under different conditions: Currently, most electride-based materials are typically limited to operating under reducing atmospheres. To broaden their applicability, it is necessary to develop electrides that can function effectively in the presence of oxidizing atmospheres or different solvent systems. First-principles calculations and machine learning have facilitated the identification of numerous electrides and the prediction of their properties, providing valuable information to screen suitable catalysts for various applications. Additionally, one could explore methods to shield electrides by applying inert coatings without compromising their unique electronic features.

4. Finding new catalytic reactions combined with external stimuli: Recently, investigating the potential synergistic effect between catalytic processes and external stimuli, such as electric or magnetic fields, has become an intriguing research topic. The unique properties of electride materials make them an excellent candidate in this area. In addition, it is worth anticipating the catalytic performance of electrides under optical or pulse excitation and their potential role in the total synthesis of bioresponsive molecules. It should be noted that research on electride-based catalysts is still in its early stages, leaving ample room for exploration.

## Declaration of competing interest

The authors declare that they have no conflicts of interest in this work.
